# Molecular docking analysis of the oral tumor target JAK STAT 3 with oxo-azo compounds

**DOI:** 10.6026/97320630019063

**Published:** 2023-01-01

**Authors:** Kaviya Karikalan, Vishnu Priya Veeraraghavan, Surya Sekaran, Gayathri Rengasamy, Kavitha Sankaran, Rajalakshmanan Eswaramoorthy

**Affiliations:** 1 Department of Biochemistry, Saveetha Dental College and Hospitals, Saveetha Institute of Medical and Technical Sciences, Saveetha University, Chennai - 600077, India; 2 Department of Biomaterials (Green lab), Saveetha Dental College and Hospital, Saveetha Institute of Medical and Technical Science (SIMATS), Saveetha University, Chennai - 600077, India

**Keywords:** JAK STAT 3, oxazole, oxo-azo derivatives, oral cancer, anticancer agents, in-silico

## Abstract

It is of interest to identify the JAK STAT 3 signaling inhibitors to abrogate tumorigenesis in oral cancer. Hence, molecular docking was performed with known oxazole compounds (1-5) and the 3D crystal structure of JAK-1 protein from *Homo sapiens* (PDB ID: 3EYG). The results show that the oxo-azo derivatives showed better interactions within the binding site of proteins. We report that compounds 1, 4 and 5 optimal binding features with JAK STAT 3.

## Background:

The JAK STAT pathway is an important oncogenic signaling cascade that consists of the Janus Kinase (JAK) family of non-receptor tyrosine kinases and the signal transducer of activator of transcription (STAT) family of transcription factors. Under physiological conditions, the ligand dependent activation of the JAK-STAT pathway is transient and tightly regulated. However, in most malignancies, STAT proteins and particularly STAT3, is aberrantly activated (tyrosine phosphorylation) in the majority of cancers [1]. However, the most common mechanism mediating STAT3 tyrosine phosphorylation in malignancies of epithelial origin is via increased/sustained IL-6 (Family)/ gp30 signaling. Indeed, the induction of IL 6 expression is positively regulated in a feed forward loop resulting in the amplification of this pathway [2]. NFkB, Notch and S1PR1 signaling are also positive regulators of IL 6 expression and are frequently co-expressed with activated STAT3 in cancers, whereas the aberrant signaling of other "oncogenic" pathways such as EGFR, HER2, Ras and Rho can also result in increased IL-6 production and subsequent STAT3 activation [3]. pSTAT3 expression and paracrine cytokine expression shows that there is growing evidence supporting the role of STAT3 in the regulation of the molecules processes shaping the tumor microenvironment as well as the function of the cells that constitute it. Immuno histo chemical and immuno fluorescent approaches used to examine the intensity, distribution and number of cells expressing activated STAT3 has revealed significant heterogeneity within the tumor stroma, as the highest pSTAT3 levels are primarily located on the leading edge of tumor in association with stromal, immune and endothelial cells [4]. Phosphorylated STAT3 expression in cells that constitute the tumor stroma is now recognized as a critical contributor to cancer pathogenesis and response to therapy pSTAT3 expression and other cell types in the tumor microenvironment [5]. There is increasing evidence that links IL-6/STAT3 to the functional properties of the cells that form the tumor microenvironment [6]. For example, contrary to normal fibroblasts, cancer associated fibroblasts (CAFs) release high levels of IL-6 and CCL2 upon STAT3 activation in co-cultured breast cancer cells, promoting the stem cell renewal and atmosphere forming capacity [7]. Attempts to find direct inhibitors of STAT3 have focused on the development of agents that target the SH2 domain in order to prevent STAT3 phosphorylation and dimerization. For example, targeting the SH2 domain of STAT3 with a novel small molecule decreased the percentage of breast cancer tumor-initiating cells as well as mammo sphere formation. The use of JAK inhibitors has been found to be more clinically effective in the treatment of myelo proliferative disorders. Additionally, JAK inhibitors (JAK 1, JAK2 and combinations) are currently in clinical trials (phase I and II) for the treatment of solid tumors. Pre-clinical studies have shown that inhibitors specific to JAK decreased the in vivo growth of a number of different cancer models. Therefore, it is of interest to document the molecular docking analysis data of the oral tumor target JAK STAT 3 with oxo-azo compounds for further consideration in drug discovery.

## Material and Methods:

## Preparation of ligands:

**Figure 1:**
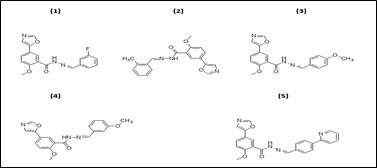
2D structure of the prepared oxo-azo compounds (1-5)

The 2D structures of the selected oxo-azo compounds (1-5) were prepared using ChemOffice suite 16.0 (Figure 1). The ligands were prepared in accordance with the standard protocol. All parameters were selected in order to achieve a stable structure with the least amount of energy. The structural optimization approach was used to estimate the global lowest energy of the title chemical. Each molecule’s 3D coordinates (PDB) were determined using optimized structure.

## Preparation of molecules:

**Figure 2:**
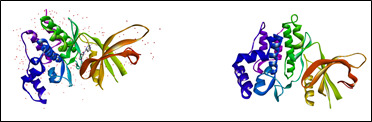
3D structure of the JAK-1 protein of *Homo sapiens*

The 3D crystal structure of the JAK-1 protein of *Homo sapiens* (PDB ID: 3EYG) was downloaded from the protein data bank (Figure 2). As per standard protocol, protein preparation was done using the software Biovia Discovery Studio and Mgl tools 1.5.7. Water molecules, co-crystallized ligands and other hetro atoms were removed and the protein was produced by adding polar hydrogens and Kollmans charges with Auto Prep.

## Molecular docking:

**Figure 3:**
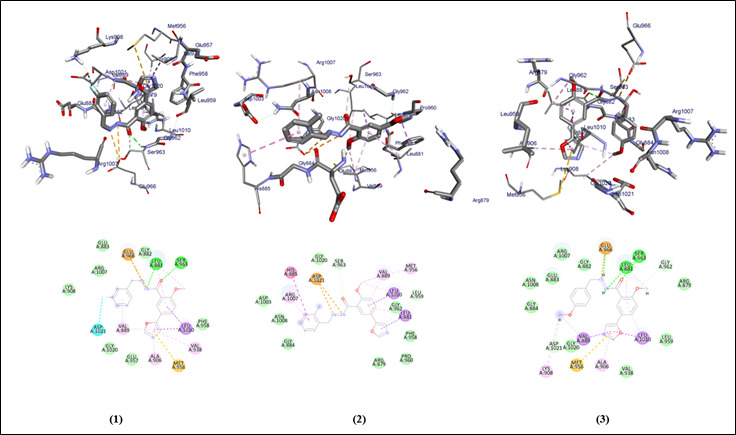
Molecular docking analysis of compounds 1, 2 and 3 against the JAK-1 protein of *Homo sapiens*

**Figure 4:**
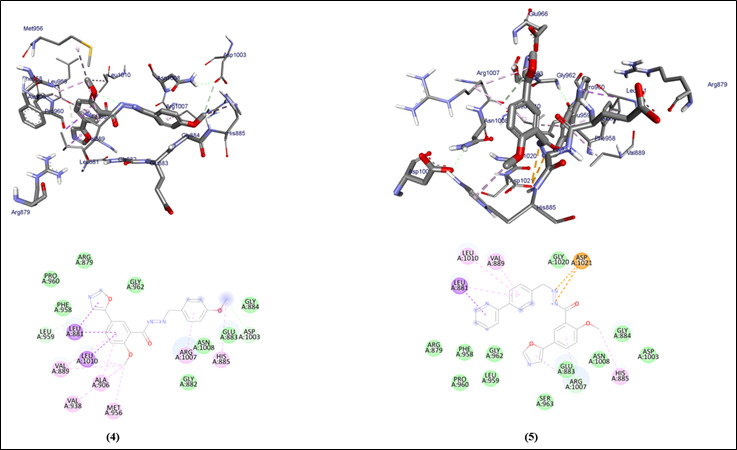
Molecular docking analysis of compounds 4, and 5 against the JAK-1 protein of *Homo sapiens*

The graphical user interface Auto Dock vina was used for Ligand-Protein docking interactions (Figure 3-Figure 4). Auto Dock Tools (ADT), a free visual user interface (GUI) for the AutoDock Vina software, was used for the molecular docking research. The grid box was built with dimensions 24.1559, 21.0952, 25.0 pointing in the x, y, and z axes. The central grid box for 3EYG was 9.8980, 13.7129, 16.6305 A. For each ligand, nine alternative conformations were created and ranked based on their binding energies utilizing Auto Dock Vina algorithms.

## In-Silico drug likeness and toxicity predictions:

SwissADME and ProTox II online servers were used to check the pharmacokinetic properties (ADME), drug-likeness, and toxicity profiles of the oxo-azo compounds (1-5). The physicochemical properties (molar refractivity, topological polar surface area, number of hydrogen bond donors/ acceptors); pharmacokinetics properties (GI absorption, BBB permeation, P-gp substrate, cytochrome-P enzyme inhibition, skin permeation (logKp)) which are critical parameters for prediction of the absorption and distribution of drugs within the body, and drug likeness (Lipinski's rule of five) were predicted using SwissADME. The toxicological endpoints (hepatotoxicity, carcinogenicity, immunotoxicity, mutagenicity) and the level of toxicity (LD50, mg/Kg) are determined using the ProTox-II server.

## Statistical analysis:

One way ANOVA was used for statistical analysis. The clinically proven drugs are used as a control and the results are compared. The significance of the results was found to be p< 0.05.

## Results:

## Molecular docking interaction of oxo-azo compounds against JAK-1 protein of *Homo sapiens*:

All the compounds (1-5) with the JAK-1 protein of *Homo sapiens* show the binding affinity ranging between -8.7 to -10 (Table 1). The compounds 1 and 3 shows hydrogen molecules interaction, and all the compounds (1-5) show hydrophobic and Van dar Waals interactions. The oxo-azo compounds have Leu-881, Ser-963, Glu-966, Val-889, and other amino acids similar to the control group Doxorubicin, Paclitaxel, and Tamoxifen within the binding site of the protein.

## SwissADME and Lipinski's rule of five:

The compounds show log Kp values between -6.05 to -6.42 cm/s (Table 2). All the compounds show high gastro intestinal absorption so it doesn't need a carrier molecule. Compounds 1, and 2 show blood brain barrier permeability. All the compounds (1-5) obey Lipinski's rule of five and are better compared to the control groups (Table 3).

## Toxicity profiling:

The compounds show class 5 toxicity (Table 4). All the compounds (1-5) show a similar LD50 value (4920 mg/kg). Compounds 1-5 are inactive in mutagenicity, and cytotoxicity.

## Discussion:

Several studies have documented that aberrant activation of the STAT3 signaling pathway contributes to neoplastic transformation in various malignancies, and have validated STAT3 as a promising target for cancer therapy [8]. The development of agents that target STAT3 with adequate potency and tumor selectivity has proven to be a difficult task. Studies by others and us have indicated that phytochemicals are involved in cancer chemoprevention by modulating the signaling circuits aberrant in cancer [9]. The functions of STAT3 protein mainly depend on its phosphorylation and subcellular localization. In unstimulated cells, the STAT3 proteins are present in the inactive form in the cytosol [10]. Activation of STAT3 occurs through phosphorylation of its tyrosine residue by cytokine or growth factor receptor signaling. Phosphorylated STAT3 then dimerizes and translocate to the nucleus where it binds to IFN-gamma-activated site (GAS) in DNA and activates the transcription of target genes. STAT-3 is found to be constitutively active in different carcinomas and inhibition of STAT-3 activation correlates with suppression of malignant cells both in vivo and in vitro. Inhibition of IL-6/JAK/STAT3 signaling can also affect the tumor microenvironment and has implications for antitumor immunity; therefore, determining whether co-targeting of immune checkpoints and the IL-6/JAK/STAT3 signaling pathway might be beneficial is important [14]. Early indications suggest that inhibition of IL-6/JAK/STAT3 signaling will be useful in combating the various adverse inflammatory effects resulting from treatment with immune-checkpoints inhibitors. Moreover, preclinical evidence is emerging that inhibition of IL-6/JAK/STAT3 signaling might augment the antitumour efficacy of immune checkpoint inhibitors [15]. Treatment of patients with cancer with immune checkpoint inhibitors can stimulate the production of IL-6 [16,17,18,19, 20]. In this present study, molecules with docking scores less than -8.5 are the lead compounds. Compared to the clinically proven drug the selected ligands have shown better interaction. The selected compounds 1, and 3 show more than two H-bonds (Leu-881, Ser-963), within the binding site indicating the stronger interactions and stable complex formation. All the Selected compounds are following Lipinski rule of 5. All the ligands show high Gastro intestinal absorption. All the ligands are skin permeable and there is no Blood Brain Barrier permeation except compounds 1, and 2. All compounds show large LD50 value and they are not cytotoxic.

## Conclusion:

Oxo-azo derivatives are shown to have better dicing interactions within the binding site of the protein. Among them, compounds 1, 4 and 5 are potentially lead molecules and act as the anti-cancer agents against JAK 1 proteins of *Homo sapiens*. They all satisfy Lipinski’s rule of five without violation which suggests that these compounds could possibly be anticancer agents. In vitro studies should be carried out to develop the molecules further.

## Abbreviation:

LD- lethal dose parameter, STAT-signal transducer of activator of transcription, JAK-Janus Kinase, CYP-cytochrome-P enzymes

## Figures and Tables

**Table 1:** Molecular docking interaction of the oxo-azo compounds (1-5) against JAK-1 protein of *Homo sapiens* (PDB ID: 3EYG)

Ligands	Docking scores/Affinity (kcal/mol)	H-bond	Amino Acid Residual interactions	
			Hydrophobic/Pi-Cation	Van dar Waals
1	-9	Leu-881, Ser-963	Glu-966, Asp-1021, Val-889, Ala-906, Met-956, Val-938, Leu-1010	Gly-882, Glu-883, Arg-1007, Lys-908, Gly-1020, Glu-957, Phe-958
2	-8.7		Ser-963, Asp-1021, His-885, Arg-1007, Val-889, Met-956, Leu-1010, Leu-959, Leu-881	Gly-1020, Asp-1003, Asn-1008, Gly-884, Arg-879, Pro-960, Phe-958, Gly-962
3	-8.8	Leu-881, Ser-963	Glu-966, Asp-1021, Lys-908, Val-889, Met-956, Ala-906, Val-938, Leu-1010, Gly-962	Gly-882, Arg-1007, Glu-883, Asn-1008, Gly-884, Gly-1020, Val-938, Val-938, Leu-959, Arg-879
4	-9.1		Leu-881, Leu-1010, Val-889, Val-938, Ala-906, Met-956, Arg-1007, His-885, Leu-959, Asp–1003	Gly-962, Arg-879, Pro-960, Phe-958, Gky-882, Asn-1008, Glu-883, Gly-884
5	-10		Asp-1021, Val-889, Leu-1010, Leu-881, His-885, Arg-1007	Gly-1020, Arg-879, Phe-958, Gly-962, Pro-960, Leu-959, Ser-963, Glu-883, Asn-1008, Gly-884, Asp-1003
Doxorubicin	-9.4	Glu-966, Asn-1008, Asp-1003	Ala-906, Val-889, Val-938, Met-956, Leu-1010, Leu-881, Gly-962, His-885, Glu-883	Phe-958, Ser-963, Gly-884
Paclitaxel	-7.7	Glu-883, Arg879	Leu-881, Leu-1010, Val-889, Ala-906, Asp-880	Asp-1021, Lys-908, Ser-963, Gly-884, Asn-1008, Leu959, Gly-962, Pro-960, Ser-961, Gly-882
Tamoxifen	-8.2	Arg-879, Lys-970	Phe-958, Leu-881, Ala-906, Leu-1010, Val-889, Ser-961	Leu-959, Gly-882, Ser-963, Gly-962

**Table 2:** SwissADME values of oxo-azo compounds (1-5)

Compound	log Kp (cm/s)	GI absorption	BBB permeant	Pgp substrate	CYP1A2 inhibitor	CYP2C19 inhibitor	CYP2C9 inhibitor	CYP2D6 inhibitor	CYP3A4 inhibitor
1	-6.25	High	Yes	No	Yes	Yes	Yes	No	No
2	-6.05	High	Yes	No	Yes	Yes	Yes	No	Yes
3	-6.42	High	No	No	Yes	Yes	Yes	No	Yes
4	-6.42	High	No	No	Yes	Yes	Yes	No	Yes
5	-6.27	High	No	No	Yes	Yes	Yes	Yes	Yes
Doxorubicin	-8.71	Low	No	Yes	No	No	No	No	No
Paclitaxel	-8.91	Low	No	Yes	No	No	No	No	No
Tamoxifen	-3.5	Low	No	Yes	No	Yes	No	Yes	No

**Table 3:** Lipinski and Veber rules of oxo-azo compounds (1-5)

Compound	MW	iLogP	HBD (nOHNH)	HBA (nON)	nrotb	MR	TPSA	Lipinski #violations	Bio availability score
Lipinski*	≤500	≤5	≤5	≤10	≤10	-	-		
Veber**	-	-	-	-	-	-	≤ 140		
1	339.32	2.45	1	6	6	89.56	76.72	0	0.55
2	335.36	2.44	1	5	6	94.57	76.72	0	0.55
3	351.36	2.42	1	6	7	96.09	85.95	0	0.55
4	351.36	2.87	1	6	7	96.09	85.95	0	0.55
5	398.41	2.48	1	6	7	112.83	89.61	0	0.55
Doxorubicin	543.52	2.16	6	12	5	132.66	206.07	3	0.17
Paclitaxel	853.91	4.51	4	14	15	218.96	221.29	2	0.17
Tamoxifen	371.51	4.64	0	2	8	119.72	12.47	1	0.55

**Table 4:** Toxicity profile of oxo-azo compounds (1-5)

			Toxicity				
Compound	^a^LD_50_ (mg/kg)	Class	HEPATOTOXICITY	CARCINOGENICITY	IMMUNOTOXICITY	MUTAGENICITY	CYTOTOXICITY
1	4920	5	ACTIVE	ACTIVE	ACTIVE	INACTIVE	INACTIVE
2	4920	5	ACTIVE	ACTIVE	ACTIVE	INACTIVE	INACTIVE
3	4920	5	ACTIVE	ACTIVE	ACTIVE	INACTIVE	INACTIVE
4	4920	5	ACTIVE	ACTIVE	ACTIVE	INACTIVE	INACTIVE
5	4920	5	ACTIVE	ACTIVE	ACTIVE	INACTIVE	INACTIVE
Doxorubicin	205	3	INACTIVE	INACTIVE	ACTIVE	ACTIVE	ACTIVE
Paclitaxel	134	3	INACTIVE	INACTIVE	ACTIVE	INACTIVE	ACTIVE
Tamoxifen	1190	4	ACTIVE	INACTIVE	ACTIVE	INACTIVE	INACTIVE
^a^LD_50_: lethal dose parameter
